# 2-Phenyl­imidazolium nitrate monohydrate

**DOI:** 10.1107/S1600536809050703

**Published:** 2009-11-28

**Authors:** Dao-Cheng Xia, Wan-Cheng Li, Shuang Han

**Affiliations:** a Yuncheng University, College of Chemistry, Yuncheng 044000, People’s Republic of China; b State Key Laboratory of Integrated Optoelectronics, Jilin University, Changchun 130021, People’s Republic of China

## Abstract

In the title hydrated mol­ecular salt, C_9_H_9_N_2_^+^·NO_3_^−^·H_2_O, the dihedral angle between the aromatic rings in the cation is 11.09 (8)°. In the crystal, the components are linked into chains propagating in [101] by N—H⋯O and O—H⋯O hydrogen bonds.

## Related literature

For related structures containing 2-phenyl­imidazole, see: Liu *et al.* (2008 ▶); Yang *et al.* (2008 ▶).
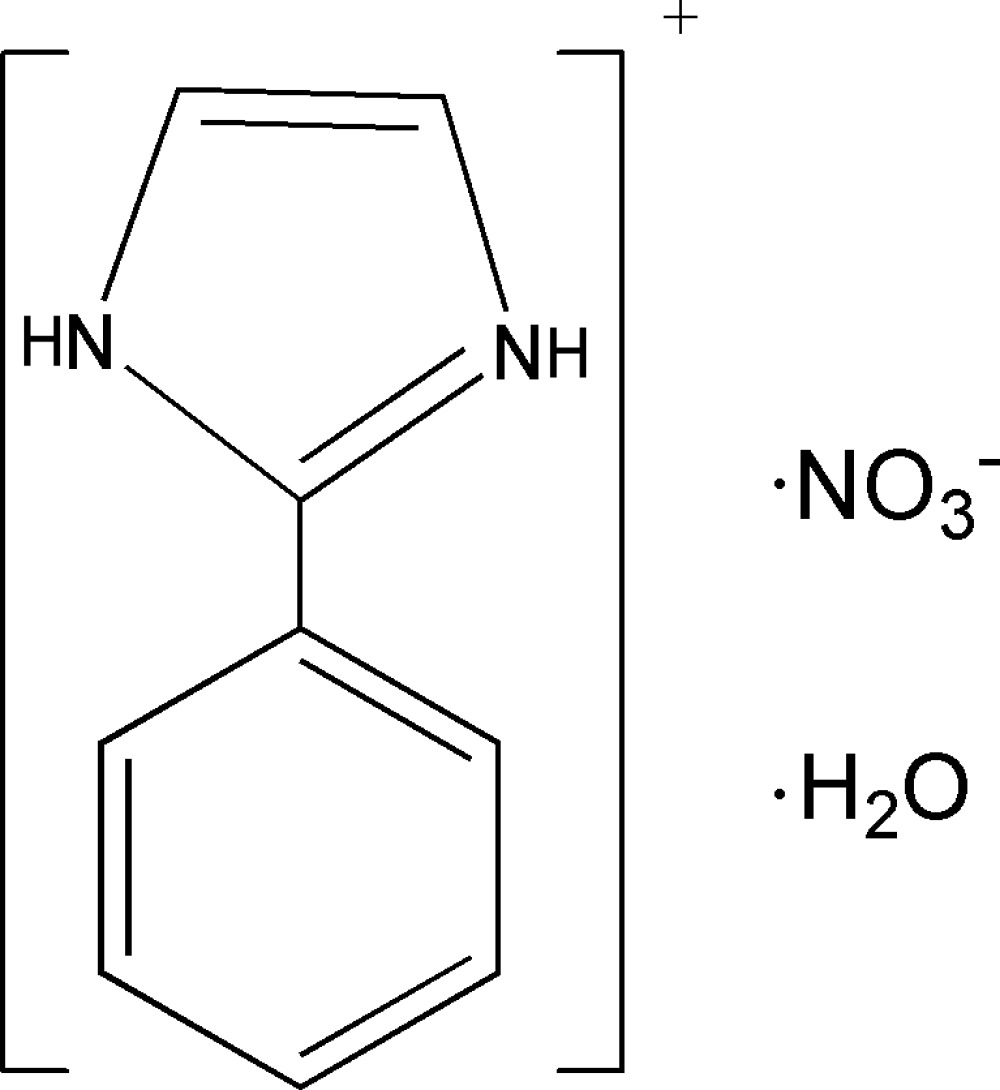


## Experimental

### 

#### Crystal data

C_9_H_9_N_2_^+^·NO_3_^−^·H_2_O
*M* *_r_* = 225.21
Monoclinic, 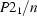

*a* = 8.026 (4) Å
*b* = 14.951 (7) Å
*c* = 8.895 (5) Å
β = 101.096 (5)°
*V* = 1047.4 (9) Å^3^
*Z* = 4
Mo *K*α radiation
μ = 0.11 mm^−1^
*T* = 293 K
0.33 × 0.28 × 0.22 mm

#### Data collection

Bruker SMART APEX CCD diffractometer
Absorption correction: multi-scan (*SADABS*; Sheldrick, 1996 ▶) *T*_min_ = 0.56, *T*_max_ = 0.81
4388 measured reflections
2407 independent reflections
1430 reflections with *I* > 2σ(*I*)
*R*_int_ = 0.018

#### Refinement

*R*[*F* ^2^ > 2σ(*F* ^2^)] = 0.040
*wR*(*F* ^2^) = 0.104
*S* = 0.88
2407 reflections
153 parameters
H atoms treated by a mixture of independent and constrained refinement
Δρ_max_ = 0.15 e Å^−3^
Δρ_min_ = −0.21 e Å^−3^

### 

Data collection: *SMART* (Bruker, 1998 ▶); cell refinement: *SAINT* (Bruker, 1998 ▶); data reduction: *SAINT*; program(s) used to solve structure: *SHELXS97* (Sheldrick, 2008 ▶); program(s) used to refine structure: *SHELXL97* (Sheldrick, 2008 ▶); molecular graphics: *SHELXTL* (Sheldrick, 2008 ▶); software used to prepare material for publication: *SHELXTL*.

## Supplementary Material

Crystal structure: contains datablocks global, I. DOI: 10.1107/S1600536809050703/hb5245sup1.cif

Structure factors: contains datablocks I. DOI: 10.1107/S1600536809050703/hb5245Isup2.hkl

Additional supplementary materials:  crystallographic information; 3D view; checkCIF report

## Figures and Tables

**Table 1:** Hydrogen-bond geometry (Å, °)

*D*—H⋯*A*	*D*—H	H⋯*A*	*D*⋯*A*	*D*—H⋯*A*
N2—H2⋯O1*W*	0.86	1.92	2.753 (2)	163
N3—H3⋯O1^i^	0.86	1.94	2.7809 (17)	166
O1*W*—H*W*11⋯O2^ii^	0.83 (2)	2.21 (2)	2.989 (2)	155.3 (19)
O1*W*—H*W*12⋯O2	0.87 (2)	2.07 (2)	2.905 (2)	162.3 (19)

Symmetry codes: (i) 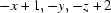; (ii) 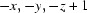.

**Fig. 1.**
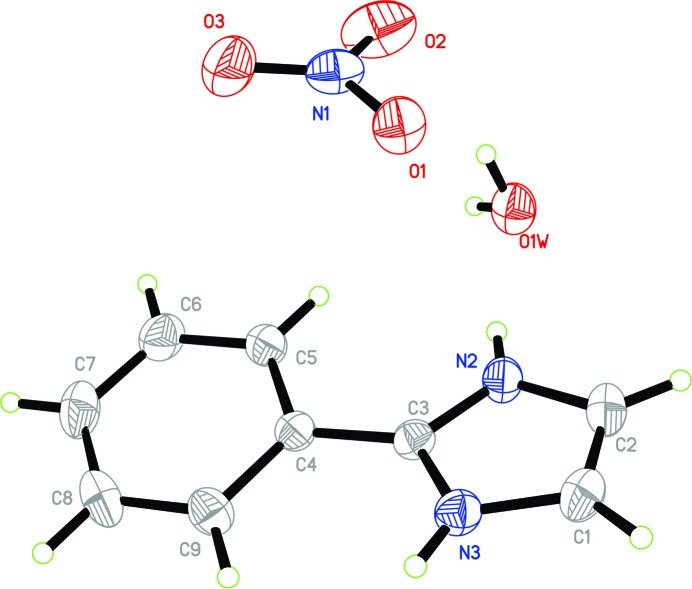
The structure of (I), showing displacement ellipsoids drawn at the 30% probability level.

## supplementary crystallographic information

### Comment

2-Phenylimidazole, as an important N-containing ligand with excellent coordinating abilities and fruitful aromatic systems, have been extensively used to build supramolecular architectures (Liu *et al.*, 2008; Yang *et al.*, 2008). We report here the synthesis and structure of the title compound, namely, C_9_H_11_N_3_O_4_ (I)

There are one 2-phenylimidazole cation, one nitrate anion and one water molecule in the asymmetric unit of the title compound, C_9_H_11_N_3_O_4_ (Fig. 1). In the crystal, molecules are linked into layer structures by N—H···O and O—H···O H-bonding interactions (Table 1).

### Experimental

A mixture of Cu(NO_3_)_2_^.^2.5H_2_O (0.5 mmol), 2-phenylimidazole (0.5 mmol), and H_2_O (30 mmol) was heated in a sealed vessel at 413 K for 2 days. After the mixture was slowly cooled to room temperature, colorless blocks of (I) were obtained (23% yield).

### Refinement

All H atoms on C and N atoms were positioned geometrically (C—H = 0.93 Å, N—H = 0.86Å) and refined as riding, with *U*_iso_(H)=1.2*U*_eq_(carrier). The water H-atoms were located in a difference map, and was refined freely.

### Crystal data

**Table:**

C_9_H_9_N_2_^+^·NO_3_^−^·H_2_O	*F*(000) = 472
*M**_r_* = 225.21	*D*_x_ = 1.428 Mg m^−^^3^
Monoclinic, *P*2_1_/*n*	Mo *K*α radiation, λ = 0.71073 Å
Hall symbol: -P 2yn	Cell parameters from 2407 reflections
*a* = 8.026 (4) Å	θ = 3.0–29.2°
*b* = 14.951 (7) Å	µ = 0.11 mm^−^^1^
*c* = 8.895 (5) Å	*T* = 293 K
β = 101.096 (5)°	Block, colourless
*V* = 1047.4 (9) Å^3^	0.33 × 0.28 × 0.22 mm
*Z* = 4	

### Data collection

**Table:**

Bruker SMART APEX CCD diffractometer	2407 independent reflections
Radiation source: fine-focus sealed tube	1430 reflections with *I* > 2σ(*I*)
graphite	*R*_int_ = 0.018
φ and ω scans	θ_max_ = 29.2°, θ_min_ = 2.7°
Absorption correction: multi-scan (*SADABS*; Sheldrick, 1996)	*h* = −10→10
*T*_min_ = 0.56, *T*_max_ = 0.81	*k* = −19→20
4388 measured reflections	*l* = −7→12

### Refinement

**Table:**

Refinement on *F*^2^	Primary atom site location: structure-invariant direct methods
Least-squares matrix: full	Secondary atom site location: difference Fourier map
*R*[*F*^2^ > 2σ(*F*^2^)] = 0.040	Hydrogen site location: inferred from neighbouring sites
*wR*(*F*^2^) = 0.104	H atoms treated by a mixture of independent and constrained refinement
*S* = 0.88	*w* = 1/[σ^2^(*F*_o_^2^) + (0.0625*P*)^2^] where *P* = (*F*_o_^2^ + 2*F*_c_^2^)/3
2407 reflections	(Δ/σ)_max_ < 0.001
153 parameters	Δρ_max_ = 0.15 e Å^−^^3^
0 restraints	Δρ_min_ = −0.21 e Å^−^^3^

### Special details

**Table:**

Geometry. All e.s.d.'s (except the e.s.d. in the dihedral angle between two l.s. planes) are estimated using the full covariance matrix. The cell e.s.d.'s are taken into account individually in the estimation of e.s.d.'s in distances, angles and torsion angles; correlations between e.s.d.'s in cell parameters are only used when they are defined by crystal symmetry. An approximate (isotropic) treatment of cell e.s.d.'s is used for estimating e.s.d.'s involving l.s. planes.
Refinement. Refinement of *F*^2^ against ALL reflections. The weighted *R*-factor *wR* and goodness of fit *S* are based on *F*^2^, conventional *R*-factors *R* are based on *F*, with *F* set to zero for negative *F*^2^. The threshold expression of *F*^2^ > σ(*F*^2^) is used only for calculating *R*-factors(gt) *etc*. and is not relevant to the choice of reflections for refinement. *R*-factors based on *F*^2^ are statistically about twice as large as those based on *F*, and *R*- factors based on ALL data will be even larger.

### Fractional atomic coordinates and isotropic or equivalent isotropic displacement parameters (Å^2^)

**Table:**

	*x*	*y*	*z*	*U*_iso_*/*U*_eq_	
C1	0.41911 (19)	−0.16151 (10)	1.11954 (17)	0.0528 (4)	
H1	0.4944	−0.1975	1.1858	0.063*	
C2	0.31545 (19)	−0.18758 (10)	0.99079 (18)	0.0532 (4)	
H2A	0.3044	−0.2452	0.9508	0.064*	
C3	0.27630 (15)	−0.04284 (9)	1.01806 (14)	0.0382 (3)	
C4	0.21609 (15)	0.04875 (9)	0.99206 (14)	0.0375 (3)	
C5	0.11719 (17)	0.07305 (9)	0.85184 (15)	0.0475 (4)	
H5	0.0869	0.0302	0.7756	0.057*	
C6	0.0641 (2)	0.15982 (10)	0.82541 (19)	0.0591 (4)	
H6	−0.0019	0.1753	0.7313	0.071*	
C7	0.1073 (2)	0.22399 (10)	0.9361 (2)	0.0626 (5)	
H7	0.0726	0.2829	0.9169	0.075*	
C8	0.2026 (2)	0.20012 (10)	1.0760 (2)	0.0609 (4)	
H8	0.2306	0.2432	1.1521	0.073*	
C9	0.25738 (17)	0.11317 (9)	1.10495 (17)	0.0502 (4)	
H9	0.3218	0.0979	1.1999	0.060*	
N1	0.31081 (15)	0.01630 (11)	0.54668 (14)	0.0575 (4)	
N2	0.22881 (14)	−0.11327 (7)	0.92928 (13)	0.0452 (3)	
H2	0.1546	−0.1123	0.8455	0.054*	
N3	0.39323 (14)	−0.07206 (7)	1.13512 (12)	0.0455 (3)	
H3	0.4446	−0.0394	1.2093	0.055*	
O1	0.39439 (12)	−0.03171 (7)	0.64925 (11)	0.0619 (3)	
O2	0.20267 (15)	−0.01926 (11)	0.44597 (13)	0.0921 (5)	
O1W	0.03846 (18)	−0.13296 (8)	0.63882 (14)	0.0607 (3)	
O3	0.33396 (17)	0.09768 (10)	0.54776 (15)	0.0860 (4)	
HW11	−0.049 (3)	−0.1037 (14)	0.606 (2)	0.090 (7)*	
HW12	0.104 (2)	−0.1081 (14)	0.584 (2)	0.097 (8)*	

### Atomic displacement parameters (Å^2^)

**Table:**

	*U*^11^	*U*^22^	*U*^33^	*U*^12^	*U*^13^	*U*^23^
C1	0.0531 (9)	0.0454 (9)	0.0587 (10)	0.0079 (7)	0.0078 (7)	0.0124 (7)
C2	0.0587 (9)	0.0372 (8)	0.0638 (10)	0.0043 (7)	0.0121 (7)	0.0023 (7)
C3	0.0371 (7)	0.0404 (7)	0.0367 (7)	−0.0022 (6)	0.0065 (5)	0.0017 (6)
C4	0.0352 (6)	0.0388 (7)	0.0394 (7)	−0.0026 (6)	0.0094 (5)	0.0004 (6)
C5	0.0537 (8)	0.0446 (8)	0.0435 (8)	0.0045 (7)	0.0076 (6)	0.0002 (6)
C6	0.0647 (9)	0.0536 (10)	0.0584 (9)	0.0137 (8)	0.0103 (7)	0.0122 (8)
C7	0.0614 (10)	0.0393 (8)	0.0896 (13)	0.0091 (7)	0.0210 (9)	0.0066 (8)
C8	0.0573 (9)	0.0459 (9)	0.0798 (12)	−0.0053 (8)	0.0140 (8)	−0.0217 (8)
C9	0.0478 (8)	0.0504 (8)	0.0502 (9)	−0.0019 (7)	0.0037 (6)	−0.0079 (7)
N1	0.0460 (7)	0.0799 (11)	0.0466 (8)	0.0057 (7)	0.0090 (6)	0.0068 (7)
N2	0.0488 (7)	0.0375 (6)	0.0461 (7)	−0.0002 (5)	0.0013 (5)	0.0004 (5)
N3	0.0459 (6)	0.0464 (7)	0.0418 (7)	0.0006 (5)	0.0020 (5)	0.0022 (5)
O1	0.0602 (7)	0.0620 (7)	0.0550 (7)	0.0060 (5)	−0.0102 (5)	0.0027 (5)
O2	0.0663 (7)	0.1416 (14)	0.0571 (8)	−0.0208 (8)	−0.0163 (6)	0.0107 (8)
O1W	0.0608 (7)	0.0538 (7)	0.0612 (8)	−0.0042 (6)	−0.0041 (6)	−0.0026 (5)
O3	0.1057 (11)	0.0655 (9)	0.0903 (10)	0.0136 (7)	0.0277 (8)	0.0200 (7)

### Geometric parameters (Å, °)

**Table:**

C1—C2	1.337 (2)	C6—H6	0.9300
C1—N3	1.3646 (19)	C7—C8	1.376 (2)
C1—H1	0.9300	C7—H7	0.9300
C2—N2	1.3678 (18)	C8—C9	1.380 (2)
C2—H2A	0.9300	C8—H8	0.9300
C3—N2	1.3274 (17)	C9—H9	0.9300
C3—N3	1.3340 (17)	N1—O3	1.2305 (19)
C3—C4	1.4556 (19)	N1—O2	1.2406 (17)
C4—C9	1.3849 (19)	N1—O1	1.2498 (16)
C4—C5	1.3917 (19)	N2—H2	0.8600
C5—C6	1.372 (2)	N3—H3	0.8600
C5—H5	0.9300	O1W—HW11	0.83 (2)
C6—C7	1.371 (2)	O1W—HW12	0.87 (2)
			
C2—C1—N3	106.90 (12)	C6—C7—H7	120.4
C2—C1—H1	126.5	C8—C7—H7	120.4
N3—C1—H1	126.5	C7—C8—C9	120.96 (14)
C1—C2—N2	106.88 (13)	C7—C8—H8	119.5
C1—C2—H2A	126.6	C9—C8—H8	119.5
N2—C2—H2A	126.6	C8—C9—C4	119.77 (14)
N2—C3—N3	106.43 (12)	C8—C9—H9	120.1
N2—C3—C4	127.10 (12)	C4—C9—H9	120.1
N3—C3—C4	126.46 (12)	O3—N1—O2	120.86 (15)
C9—C4—C5	118.94 (13)	O3—N1—O1	120.21 (14)
C9—C4—C3	120.88 (12)	O2—N1—O1	118.92 (17)
C5—C4—C3	120.18 (12)	C3—N2—C2	109.92 (11)
C6—C5—C4	120.35 (13)	C3—N2—H2	125.0
C6—C5—H5	119.8	C2—N2—H2	125.0
C4—C5—H5	119.8	C3—N3—C1	109.86 (12)
C7—C6—C5	120.78 (15)	C3—N3—H3	125.1
C7—C6—H6	119.6	C1—N3—H3	125.1
C5—C6—H6	119.6	HW11—O1W—HW12	98.1 (18)
C6—C7—C8	119.18 (14)		

### Hydrogen-bond geometry (Å, °)

**Table:**

*D*—H···*A*	*D*—H	H···*A*	*D*···*A*	*D*—H···*A*
N2—H2···O1W	0.86	1.92	2.753 (2)	163
N3—H3···O1^i^	0.86	1.94	2.7809 (17)	166
O1W—HW11···O2^ii^	0.83 (2)	2.21 (2)	2.989 (2)	155.3 (19)
O1W—HW12···O2	0.87 (2)	2.07 (2)	2.905 (2)	162.3 (19)

Symmetry codes: (i) −*x*+1, −*y*, −*z*+2; (ii) −*x*, −*y*, −*z*+1.
